# Hamstrings and Quadriceps Muscles Function in Subjects with Prior ACL Reconstruction Surgery

**DOI:** 10.3390/jfmk3040056

**Published:** 2018-11-20

**Authors:** Jamie L. Faxon, Adeola A. Sanni, Kevin K. McCully

**Affiliations:** Department of Kinesiology, University of Georgia, 330 River Road, Athens, GA 30602, USA

**Keywords:** endurance, fatigue, skeletal muscle, knee rehabilitation

## Abstract

Background: As the knee joint is a common site for injury among younger people, the purpose of this study was to measure the skeletal muscle endurance and strength on people with prior anterior cruciate ligament (ACL) knee reconstruction surgery. Method: Young healthy female subjects who reported having knee reconstruction surgery more than one-year prior were tested. The skeletal muscle endurance index (EI) of the hamstrings and quadriceps muscles was determined as the decline in the specific muscle acceleration in response to 2 Hz, 4 Hz, and 6 Hz electrical stimulation. Maximal isometric muscle strength (MVC) was measured in the hamstrings and quadriceps muscles. Results: The hamstrings muscles in the injured leg had less endurance than the non-injured leg at 6 Hz stimulation (55.5 ± 13.2% versus 78.0 ± 13.3%, *p* < 0.01). Muscle endurance was not reduced in the quadriceps muscles in the injured leg compared to the non-injured leg at 6 Hz stimulation (78.0 ± 13.3% versus 80.3 ± 10.0%, *p* = 0.45). There were no differences in MVC between the injured and non-injured legs for either the hamstrings (*p* = 0.20) or quadriceps muscles (*p* = 0.67). Conclusions: Muscle endurance was reduced in the hamstrings muscles at least one-year post injury, while hamstrings strength was recovered. Reduced hamstrings muscle endurance could be a result of lack of endurance training during rehabilitation. This may contribute to re-injury in the muscle, even in people who have recovered muscle strength.

## 1. Introduction

The knee joint is a common site for injury among younger people, and muscle weakness commonly persists in individuals who return to activity following anterior cruciate ligament (ACL) reconstruction [1,2]. People are more likely to suffer from an injury when they spend their leisure time practising sporting activities. A 10-year study documented 17,397 patients with several sports injuries including knee injuries [3]. The ACL was damaged in 45.4% of the cases of internal knee injuries [3]. Female athletes report ACL injuries at a 4-to-6-fold greater rate than male athletes [4,5]. Moreover, many athletes do not successfully return to their pre-injury sport, despite reaching the acceptable requirements for muscle function [6].

Rigorous rehabilitation after ACL reconstruction is required for a successful outcome [7], recent research suggests that there is a low rate of return to play and a high rate of re-injury [8]. This may be a result of building physical rehabilitation protocols around sports; athletes are primarily focused on returning to their desired sport, so the process of regaining muscle strength is accelerated [7]. Several studies support the claim that physical therapy emphasizes the importance of muscle strength [9,10,11]. In contrast, muscle endurance is not a focus of physical therapy following a knee injury. Impaired muscle endurance can lead to lingering weakness when playing a sport or in everyday activities. If the hamstrings muscles have reduced endurance compared to the quadriceps muscles, muscle weakness and imbalance between the two muscles could develop while performing sustained exercise. This muscle imbalance, particularly in the hamstrings muscles, may be associated with a lack of muscle endurance training in rehabilitation following an ACL knee injury.

The purpose of this study was to measure the muscle endurance in the hamstrings and quadriceps muscles following reconstructive surgery for an ACL knee injury. A noninvasive measure of muscle-specific endurance was developed to allow testing of muscle fatigue in the hamstrings and quadriceps muscles independent from motor activation [12,13,14]. It was hypothesized that subjects who have undergone reconstructive surgery and physical therapy will have impaired hamstrings muscle endurance in their injured leg compared to their non-injured leg. This is potentially due to a lack of focus on endurance training.

## 2. Materials and Methods

### 2.1. Study Population

Eight female subjects were tested. Relevant participant demographics are shown in Table 1. The subjects reported an ACL rupture via physical activity and subsequent surgical reconstruction. Each subjects’ ACL was replaced by a substitute graft made of tendon. Autografts (bone-patella tendon-bone, hamstrings, and quadriceps) and synthetic grafts were all included in the study. Each subject completed physical therapy at least 12 months prior to testing. All subjects had resumed a recreationally active lifestyle. This study was approved by the University of Georgia, Athens Institutional Review Board, and each subject signed an informed consent form before participating in the study.

### 2.2. Experimental Protocol

This study involved a one group design where both hamstrings and both quadriceps muscles were tested in each subject. Each subject was tested in a single day. The subjects were asked to report their recent activity level including the sporting activities they perform on a weekly basis. During testing, subjects participated in an endurance test on their hamstrings and quadriceps; this was done on the injured and non-injured leg. Following the endurance test, the same muscles were tested for isometric muscle strength with an isokinetic ergometer (Biodex) on each subject.

### 2.3. Endurance Test

A muscle-specific endurance test was performed as reported previously [12]. The subjects were in a prone position (for hamstrings measurement) and supine position (for quadriceps measurement) on a padded table. Using double-sided tape, a triaxial accelerometer (WAX-3, Axivity, Newcastle upon Tyne, UK) was attached to the surface of the subject’s skin; this was placed on the belly of each muscle group (hamstrings/quadriceps). Two electrodes 4 × 5 cm were placed 2 to 3 cm proximal and distal to the accelerometer across the muscles tested at a time. The muscles were stimulated using current levels between 30 and 50 mA. The stimulation current was based on the visibility of muscle twitch and comfort level of each subject. Previous studies have shown the Endurance Index measurements to be independent of the current level [12]. The triaxial accelerometer was set to collect data at 400 Hz. Stimulation consisted of 2 Hz, 4 Hz, and 6 Hz for three minutes. Each stimulation had ten seconds of rest in between each frequency and a 30 s baseline established before and after each interval of study. Declines in the acceleration of the muscle twitch contractions were used to visualize fatigue and calculate muscle endurance.

### 2.4. Muscle Strength

A Biodex System 4 Quick Set (Biodex System 4 Pro Quick Set, Biodex Medical Systems, Inc., New York, NY, USA) was used to measure the isometric muscle strength of the subject’s hamstrings and quadriceps [15]. The subjects were instructed to sit in the chair. Their knee was placed at 90 degrees with their leg strapped to a lever that measured the torque (Newton-meters). Hamstrings strength was measured during isometric knee flexion and quadriceps strength was measured during isometric knee extension. A total of six measurements were collected; three for the quadriceps and three for the hamstrings. The average of the three values was recorded as a single strength value for each muscle group. This was done on both legs.

### 2.5. Data Analysis

**Figure 1:**
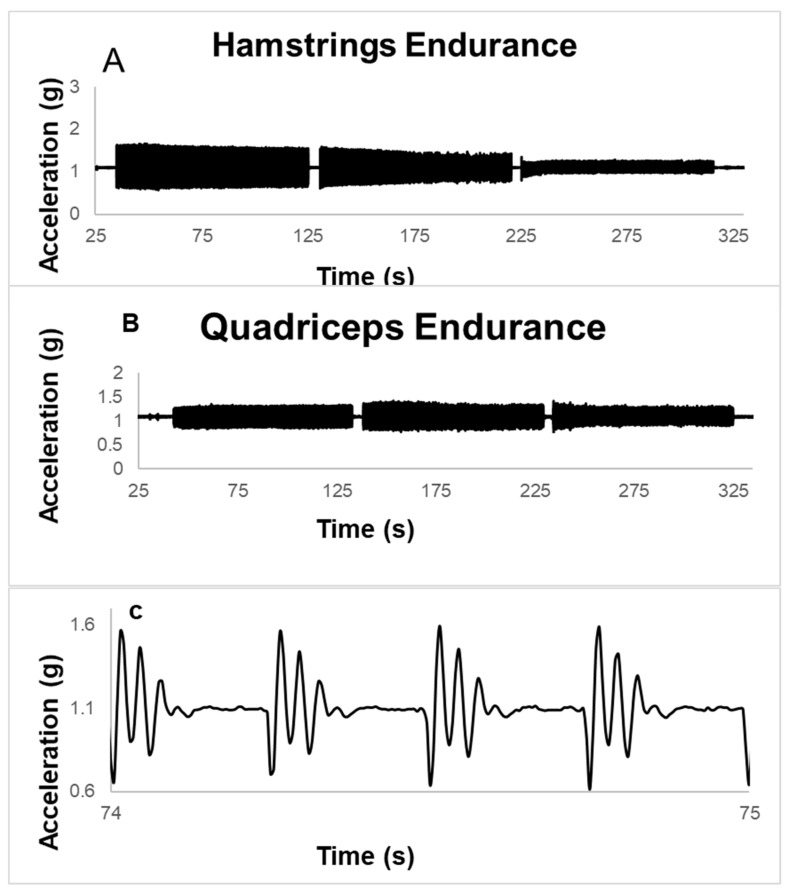
(**A**) A representative example of hamstrings endurance of the injured leg at 2 Hz, 4 Hz, and 6 Hz stimulation. (**B**) A representative example of quadriceps endurance of the injured leg at 2 Hz, 4 Hz, and 6 Hz stimulation. (**C**) Example of peak to peak twitch acceleration zoomed in one second at 4 Hz.

Data from the accelerometer was transferred to Microsoft Excel, and a resultant vector was calculated from the three axes (*X*, *Y*, and *Z*). Further analysis was done in MATLAB R2017b (Mathworks Inc., Natick, MA, USA) using a customized written analysis program. Peak to peak analysis was employed to determine the magnitude of acceleration for each contraction frequency (Figure 1C). Endurance Index data was calculated as the percent of acceleration at the end of each stimulation frequency in relation to its peak value [12,14].

### 2.6. Statistical Analysis

A two-way between and within-subject analysis of variance (ANOVA) was used to evaluate the difference between the injured and non-injured leg and the effect of the three frequencies of stimulation (2, 4, and 6 Hz). This was done for both hamstrings and quadriceps muscles. A two-way ANOVA was also used to evaluate the difference between the hamstrings and the quadriceps, as well as the effect of the stimulation frequency for each leg. A follow up pairwise comparison was done for each pair of the stimulation frequency. A 2 × 2 ANOVA was conducted to evaluate the difference between the strength of the non-injured and the injured leg for both the hamstrings and the quadriceps muscles. Significance was accepted at an alpha level ≤0.05.

## 3. Results

Data were obtained from eight females with prior ACL knee injuries. (The average time after the injury was 54 months (ranging between 29 to 94 months.) Table 1 shows the descriptive statistics of the participants. Figure 1A (hamstrings) and 1B (quadriceps) show the representative figure of the endurance graph of one of the participants at the three frequencies in absolute value (*g*).

### 3.1. Hamstring Endurance

**Figure 2:**
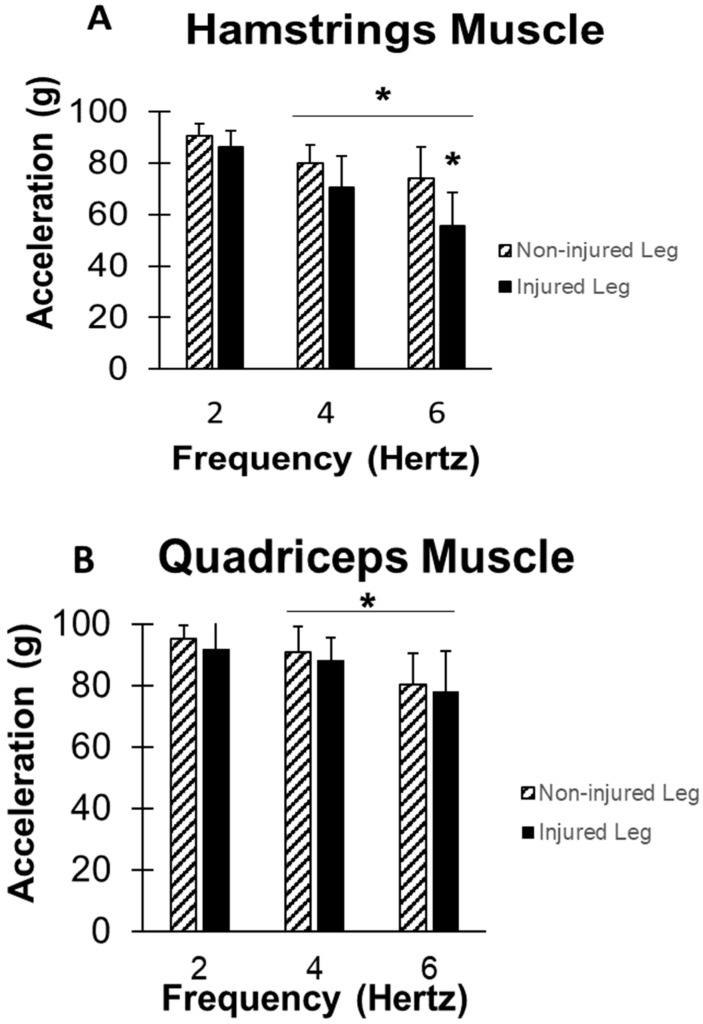
(**A**) Mean and standard deviation of the endurance index for the hamstrings muscle at three frequencies (2 Hz, 4 Hz, and 6 Hz) for each leg. (**B**) Mean and standard deviation of the endurance index of the quadriceps muscle at three frequencies (2 Hz, 4 Hz, and 6 Hz) for each leg. * represents significant difference (*p* < 0.05).

Figure 2A shows the mean and standard deviation of the endurance index of the participants for non-injured hamstrings and injured hamstrings, respectively. The hamstrings muscles in the injured leg had lower endurance than the non-injured leg at 2 Hz (86.3% ± 6.3% versus 90.5% ± 4.9%), 4 Hz (70.5% ± 12.2% versus 80.1% ± 6.9%), and 6 Hz stimulation (55.5% ± 13.2% versus 73.9% ± 12.3%), (F(1,14) = 11.45, *p* < 0.01). There was also a significant difference in the effect of the frequency (F(2,28) = 26.26, *p* < 0.001).

### 3.2. Quadriceps Endurance

Figure 2B shows the mean and standard deviation of the Endurance Index of the participants for non-injured quadriceps and injured quadriceps, respectively. ANOVA shows no significant difference in the Endurance Index of the quadriceps muscle between the injured and the non-injured leg (F(1,14) = 0.609, *p* = 0.448). Although, the within-subjects shows a significant effect of frequency (F(2,28) = 14.80, *p* < 0.01). Mixed-design ANOVA shows that there was a significant difference in Endurance Index between the hamstrings and the quadriceps of the non-injured leg (F(1,14) = 7.794, *p* < 0.01) and injured leg (F(1,14) = 13.536, *p* < 0.01), respectively. Within-subject ANOVA shows a significant effect of the stimulating frequency (F(2,28) = 15.330, *p* < 0.01) and (F(2,28) = 24.896, *p* < 0.01), respectively.

### 3.3. Muscle Strength

**Figure 3:**
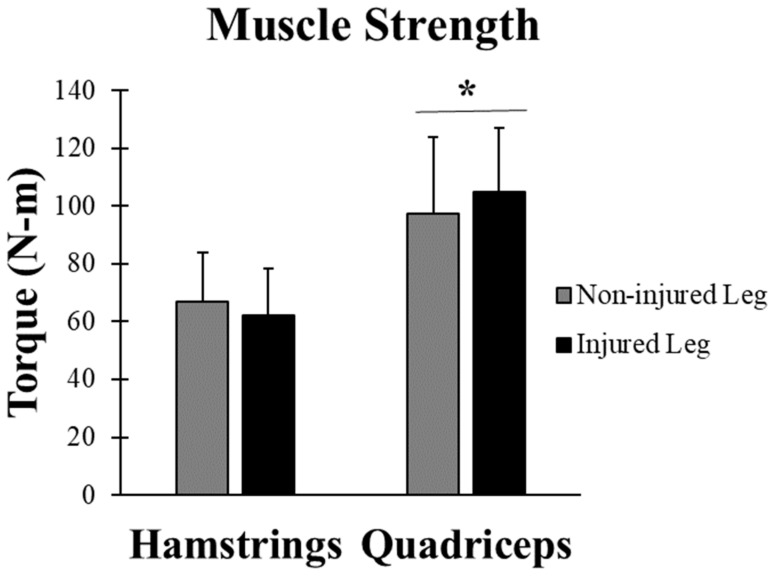
The muscle strength of the hamstrings and quadriceps. There was no significant difference between the injured and non-injured legs (*p* > 0.05), but there was a significant difference between the hamstrings and the quadriceps (*p* < 0.05). Values are means (SD). * represents significant difference (*p* ≤ 0.05).

The mean and standard deviation of the muscle strength of both hamstrings and quadriceps are shown in Figure 3. There was no significant difference in muscle strength between the injured and non-injured leg based on a 2 × 2 ANOVA shows (F(1,28) = 0.027, *p* = 0.87). There was a significant difference between the muscle strength of the hamstrings and the muscle strength of the quadriceps muscles (F = 1,28) = 24.84, *p* < 0.01) of both injured and non-injured legs. The hamstrings:quadriceps (H:Q) ratio was calculated using the averages for the injured and non-injured legs, these values (injured = 0.59) and (non-injured = 0.69) fell in the normal range of 0.50 to 0.80 [12].

## 4. Discussion

The main finding of this study is that the hamstrings endurance in the injured leg was reduced in individuals with prior ACL reconstruction surgery. Other studies have evaluated the relationship between fatigue and ACL injuries, but few studies focus directly on muscle endurance of the hamstrings following ACL reconstructive surgery. A study found that the inability to fully activate the quadriceps muscles following ACL surgery is common and proposed that endurance training may restore normal quadriceps function [16]. While this research highlights the importance of endurance training, it focused more on how the improper function of the quadriceps can lead to hamstrings muscles impairment. Our study, however, shows that the quadriceps might have fully recovered both strength and endurance, while the hamstrings muscles were not fully recovered in endurance. Another study found quadriceps muscles endurance was reduced compared to controls 18 months after surgery, with no difference in hamstrings muscles endurance at the same time [17]; the endurance protocol was performed with an isokinetic ergometer at various muscle speeds. That study did not make a comparison with the non-injured leg. Another study found no differences in hamstrings or quadriceps muscles endurance 26 months following injury [18]; the endurance test involved total knee flexion work performed in 45 s using an isokinetic ergometer. The endurance protocol in our study is distinct because it did not rely on voluntary muscle contractions.

Previous studies have reported long-term deficits in perceived and functional outcomes which might be related to central factors. Central factors do not play a role in the endurance test used in this study because of the use of electrical stimulation to produce muscle contractions [12]. Our results are consistent with a previous study that reported reduced trunk muscle endurance at least one year following ACL reconstruction surgery [17]. Werner et al. concluded that inadequate rehabilitation of the trunk muscles was the reason for the deficit in trunk muscle endurance [19].

In this study, we found that hamstrings and quadriceps muscles isometric strength were similar in the injured and non-injured legs. Previous studies have reported strength deficits in the injured leg for both the hamstrings and quadriceps muscles [20]. However, some of these deficits are not apparent 2–3 years post-injury [21]; this is consistent with our results. We found the ratio of quadriceps to hamstrings muscles strength to be within the range expected of healthy uninjured people. This suggests that our participants did recover their strength even if the hamstrings muscles endurance did not recover [22].

Our study had a few limitations. We made our measurements an average of 4.5 years after the injury. This is longer than most studies that look at muscle function post-injury. Our study does not allow us to make any conclusions on the potential time course of changes in muscle endurance, relative to changes in muscle strength. In this study, we did not correct by type of surgery to repair the injured knee. Previous studies have suggested that there is no “best” graft for ACL reconstruction [23]. The ideal graft is biomechanically similar to the native ligament, easily harvested, and incorporates well with bone [24]. The different types of grafts, however, may influence the recovery of muscle endurance and strength, and this could be a topic of interest for further research. This study only focused on female subjects, in part due to the available research subject pool [17]. As previously stated, females are more likely than males to suffer from a knee injury, most specifically a torn ACL [5,25,26,27,28]. The sex of the subject may also play a role in their recovery following reconstructive surgery, but more research remains in this area [29]. Additionally, we did not look specifically at rehabilitation because we tested recreational athletes where rehabilitation was up to the individual. On average, the subjects still expressed a deficit in muscle endurance years post-surgery. This study was not prospective, so we cannot conclude that weakness leads to injury. However, athletes with hamstrings to quadriceps ratios below the normal range are more likely to sustain an overuse injury. Therefore, balancing muscle endurance may work to reduce the likelihood of injury or re-injury [30]. Finally, this study included a relatively small sample size (*n* = 8). While our study found significant differences in hamstrings muscles endurance in the previously injured leg, additional studies will be needed to confirm and extend our findings.

This research is relevant because following the ankle, the knee joint is the second most commonly injured body part, and knee injuries are the leading cause of sport-related surgeries [31]. The effectiveness of rehabilitation and full recovery following a sustain knee injury are largely based on literature [32]. Therefore, these findings have the potential to transform rehabilitation following ACL reconstructive surgery by including an emphasis on rehabilitation of muscle endurance training in addition to traditional strength training.

## 5. Conclusion

Muscle endurance was reduced in the hamstrings muscles at least one-year post injury, while hamstrings muscle strength was not reduced. The hamstrings muscles of the injured leg of the individual did not appear to recover fully at least in terms of endurance following rehabilitation of an ACL knee injury. Quadriceps endurance, hamstring strength, and quadriceps strength did appear to recover. Reduced hamstrings muscles endurance could be due to lack of focus on muscle endurance during rehabilitation after injury; this may contribute to re-injury in the particular muscle even in people who have recovered muscle strength. Additional studies, and perhaps a focus on muscle endurance during rehabilitation, are needed to improve physical therapy and rehabilitation following knee injuries.

## Figures and Tables

**Table 1:** Relevant participant demographics.

Subject	Gender	Age (yr)	Weight (kg)	Height (cm)	BMI (kg·m^−2^)	Time After Surgery (months)	Graft Type	Previous Physical Activity
1	F	19	61.2	162.6	23.2	29	Quadriceps	Occasional rock climbing
2	F	21	65.8	167.6	23.4	59	Hamstrings	Running 4×/week + weights
3	F	21	65.8	160.0	25.7	39	Cadaver	Running 4×/week + weights
4	F	21	63.5	167.6	22.6	36	Patella Tendon	Elliptical 4×/week + weights
5	F	21	55.3	170.2	19.1	48	Hamstrings	Running 4×/week + weights
6	F	22	56.7	154.9	23.6	94	Hamstrings	Running 5×/week + weights
7	F	21	69.9	170.2	24.1	84	Hamstrings	Cycle 2×/week, soccer 1×/week. + weights
8	F	31	64.4	167.6	22.9	43	Cadaver	2mi 2×/week + weights
Mean ± SD		22.1 ± 3.7	62.8 ± 4.9	165. ± 5.4	22.1 ± 3.7	54 ± 23.5		

Notes. Time after surgery—months between the date of surgery and date tested in the research lab.
